# Osteoclast-derived IGF1 induces RANKL production in osteocytes and contributes to pagetic lesion formation

**DOI:** 10.1172/jci.insight.159838

**Published:** 2023-07-24

**Authors:** Kazuaki Miyagawa, Hirofumi Tenshin, Patrick L. Mulcrone, Jesus Delgado-Calle, Mark A. Subler, Jolene J. Windle, John M. Chirgwin, G. David Roodman, Noriyoshi Kurihara

**Affiliations:** 1 Division of Hematology and Oncology, Department of Medicine, Indiana University, Indianapolis, Indiana, USA.; 2 Department of Physiology & Cell Biology, Winthrop P. Rockefeller Cancer Institute, University of Arkansas for Medical Sciences, Little Rock, Arkansas, USA.; 3 Department of Human and Molecular Genetics, Virginia Commonwealth University, Richmond, Virginia, USA.; 4 Research Service, Roudebush Veterans Administration Medical Center, Indianapolis, Indiana, USA.

**Keywords:** Bone Biology, Endocrinology, Osteoclast/osteoblast biology

## Abstract

We previously reported that measles virus nucleocapsid protein (MVNP) expression in osteoclasts (OCLs) of patients with Paget disease (PD) or targeted to the OCL lineage in *MVNP*-transgenic mice (*MVNP* mice) increases IGF1 production in osteoclasts (OCL-IGF1) and leads to development of PD OCLs and pagetic bone lesions (PDLs). Conditional deletion of *Igf1* in OCLs of *MVNP* mice fully blocked development of PDLs. In this study, we examined whether osteocytes (OCys), key regulators of normal bone remodeling, contribute to PD. OCys in PDLs of patients and of *MVNP* mice expressed less sclerostin, and had increased RANKL expression compared with OCys in bones from WT mice or normal patients. To test whether increased OCL-IGF1 is sufficient to induce PDLs and PD phenotypes, we generated TRAP-*Igf1* (T-*Igf1*) transgenic mice to determine whether increased IGF1 expression in the absence of MVNP in OCLs is sufficient to induce PDLs and pagetic OCLs. We found that T-*Igf1* mice at 16 months of age developed PD OCLs, PDLs, and OCys, with decreased sclerostin and increased RANKL, similar to *MVNP* mice. Thus, pagetic phenotypes could be induced by OCLs expressing increased IGF1. OCL-IGF1 in turn increased RANKL production in OCys to induce PD OCLs and PDLs.

## Graphical abstract

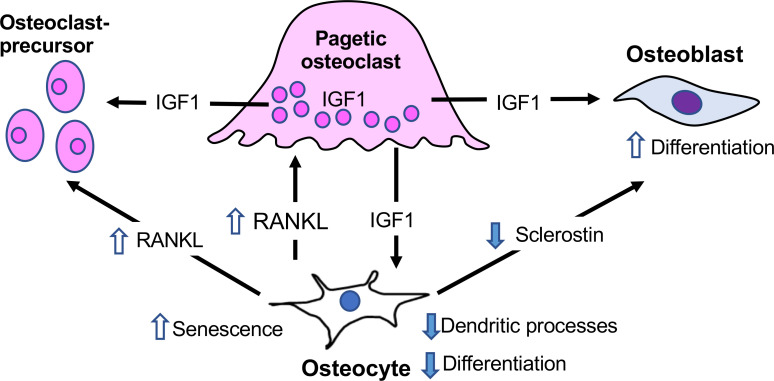

## Introduction

Paget disease (PD) of bone usually occurs in patients over 50 years of age and represents the most exaggerated example of coupled bone remodeling (1). Patients with PD have characteristic pagetic lesions (PDLs) that are highly localized areas in bone, where both osteoclast (OCL) and osteoblast (OB) activities are markedly increased. This results in local overproduction of poor-quality bone that can cause significant clinical problems (1). Interestingly, patients with PD rarely develop new focal lesions over the course of their disease. The primary cellular abnormality in PD resides in the osteoclast (2), because highly effective PD treatments that target OCL activity, such as zoledronic acid, also normalize the rapid bone formation (3).

We previously reported that OCLs from 70% of PD patients we tested express measles virus nucleocapsid protein (MVNP) (4), and transgenic mice more than 12 months old with *MVNP* targeted to OCLs (*MVNP* mice) develop PDLs and abnormal OCLs characteristic of PD (5, 6). We found that OCLs from patients with PD, but not normal donors, express elevated levels of IL-6 and IGF1. Similarly, *MVNP* expression in OCLs of PD patients also induces high levels of IL-6 in PD-OCLs (7), which in turn increases IGF1 expression in OCLs in an autocrine manner. The OCL-derived IGF1 (OCL-IGF1) then upregulates expression of the coupling factors Ephrin B2 (EphB2) and EphB4 on OCLs and OBs, respectively (8, 9). Importantly, conditional deletion of *Igf1* in OCLs of *MVNP* (*MVNP/Igf1*-cKO) mice totally blocked development of PDLs and the abnormal OCLs and bone remodeling characteristic of PD (9). These results suggest that increased expression of IGF1 in OCLs is needed for the development of PD OCLs and PDLs in vivo.

Although much is known about abnormal OCL and OB activity in PD, little is known about the mechanisms responsible for the focality and persistence of bone lesions in PD. The persistence of solitary lesions in patients with PD suggests cellular imprinting for which local modification of resident cells, such as osteocytes (OCys) within the pre-PD niche, provides an attractive mechanism. OCys are the most abundant and long-lived cells in bone, derived from late OB lineage cells that become imbedded within mineralized matrix and intercommunicate via neurite-like extensions between cells within bone canaliculi. OCys are critical regulators of local bone remodeling, the primary source of receptor activator of NF-κB ligand (RANKL) in adult bone, and producers of and responders to IGF1 (10). However, little is known about their contributions to PD. Here we report that OCys are abnormal in PD. We generated mice expressing *Igf1* under the control of the tartrate-resistant acid phosphatase (*TRAP*) promoter (T-*Igf1* mice) to test whether high levels of OCL-IGF1 alone are sufficient to induce PDLs. T-*Igf1* mice developed PDLs and pagetic OCLs and OCys at 16 months of age. Furthermore, OCys from *MVNP* and T-*Igf1* mice secreted high amounts of RANKL and low sclerostin in response to OCL-IGF1, suggesting a mechanism by which high IGF1 produced by PD OCLs promotes a positive feedback loop between OCLs and OCys, leading to development of PDLs.

## Results

### OCys in MVNP mice and patients with PD are abnormal.

To characterize the effects of increased OCL-derived IGF1 on OCys in PD, we analyzed sclerostin expression in bone sections of femurs from 20-month-old WT, *Igf1-*cKO, *MVNP*, and *MVNP/Igf1*-cKO mice.

**Figure 1:**
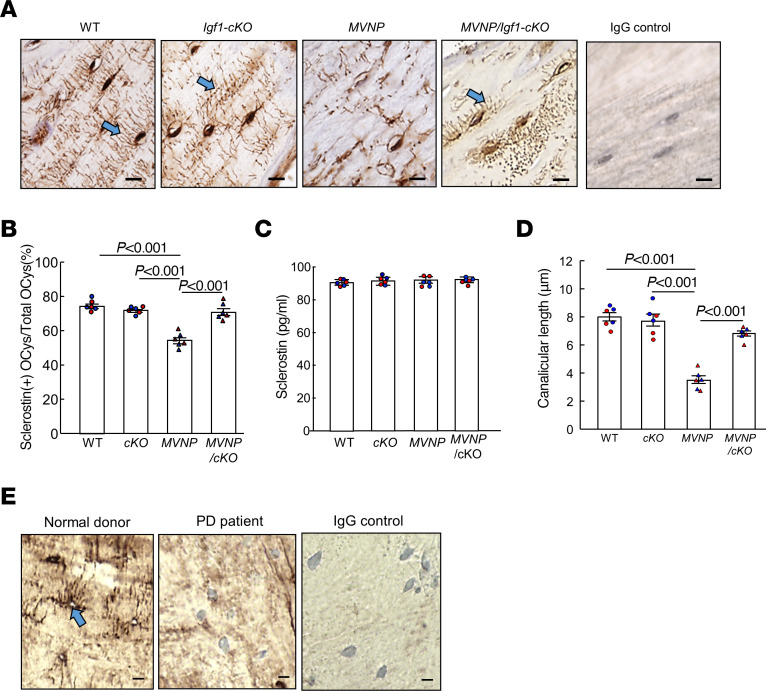
Osteocyte morphology and sclerostin expression in WT, *Igf1*-cKO, *MVNP*, and *MVNP/Igf1*-cKO mice at 20 months of age and a PD and normal patient. (**A**) Sclerostin expression was determined by immunostaining as described in Methods. Arrows indicate canalicular tubes. Scale bars: 10 μm. (**B**) Ratio of sclerostin-positive OCys/total OCys per area (0.5 mm^2^). Results are expressed as the mean ± SEM from 3 male (blue) and female (red) mice of the 4 genotypes, analyzed using a 1-way ANOVA with Tukey’s test. (**C**) Serum sclerostin from mice in **B** measured by ELISA, expressed as mean ± SEM. (**D**) Canalicular length per 0.5 mm^2^ was measured using ImageJ software from the same sections shown in **A** and used for **B**. Results are expressed as the mean ± SEM from 1 average value per mouse (randomly selected 30 measurements from each mouse) of 3 male (blue) and female (red) mice of the 4 genotypes, analyzed using a 1-way ANOVA with Tukey’s test. The same mice were used in **A**–**D**. No statistical differences were found for results between males and females. (**E**) OCy phenotype and sclerostin expression in bone specimens from a patient with Paget disease (PD) and a normal donor. Transiliac crest bone biopsies were taken from a 58-year-old female with PD (center panel) and a 30-year-old healthy female control (left) and immunostained for sclerostin. The PD patient sample was also stained with control IgG (right). Scale bars: 10 μm.

OCys in *MVNP* mice showed lower sclerostin staining compared with the other genotypes (Figure 1A), and the numbers of sclerostin-expressing OCys per bone area were also significantly reduced compared with the other genotypes (Figure 1B). However, circulating serum sclerostin concentrations were similar in all genotypes (Figure 1C). OCys in *MVNP* mice were morphologically abnormal and had very short dendritic processes compared with the well-developed dendritic processes of OCys in WT, *Igf1*-cKO, and *MVNP/Igf1-*cKO mice (Figure 1A, arrow). Canalicular length of OCys in *MVNP* mice was also significantly shorter than in the other genotypes (Figure 1D, *P* < 0.001), but did not appear to be sexually dimorphic. Importantly, OCys in a bone biopsy from a patient with PD were similarly abnormal and showed reduced sclerostin expression and shorter dendritic processes compared with OCys in a bone biopsy of a normal patient (Figure 1E, arrow).

### OCy morphology and sclerostin expression in PDLs.

**Figure 2:**
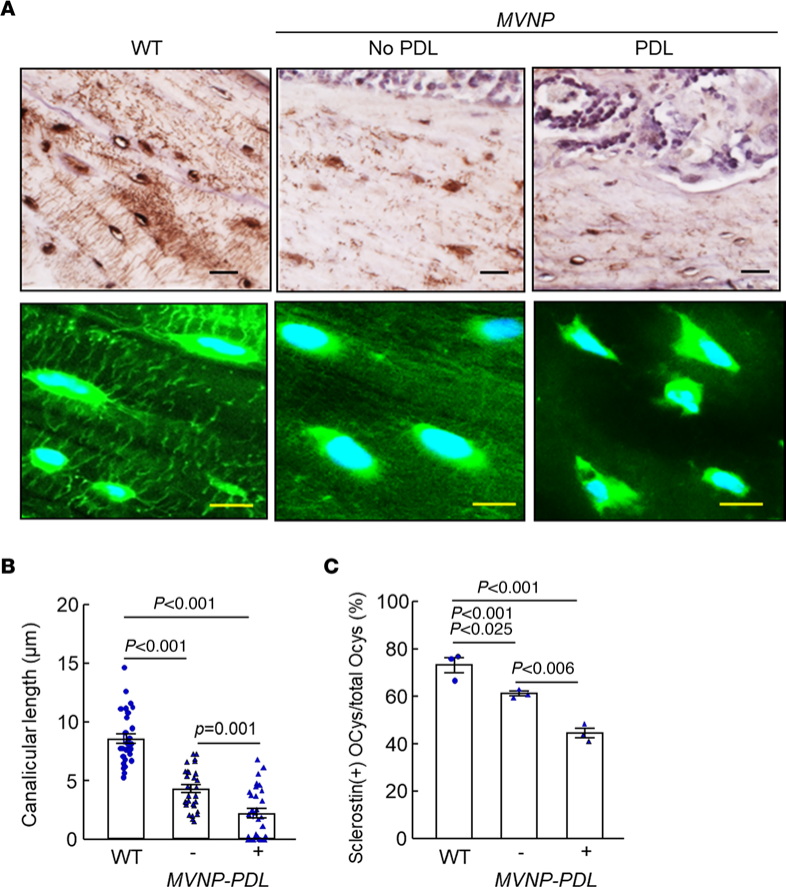
Changes in osteocyte morphology and sclerostin expression within pagetic lesions of *MVNP* mice. (**A**) OCy phenotype (upper panels, sclerostin immunostaining as in Figure 1A, scale bars: 20 μm) and Alexa Fluor 488–phalloidin fluorescent staining for F-actin of sections adjacent to those above to show osteocyte dendritic processes (lower panels, scale bars: 5 μm) in bone specimens from 20-month-old WT and *MVNP* mice with and without PD lesions (PDLs). (**B**) Canalicular length per 0.5 mm^2^ was measured as in Figure 1D. (**C**) Numbers of sclerostin-positive OCys per 100 randomly counted OCys in 3 biological replicates from WT and *MVNP* mice, shown as mean ± SEM, analyzed by 1-way ANOVA with Tukey’s test.

We and others previously found that OCLs are increased in number and hypermultinucleated in PDLs (1–3). Therefore, we examined OCys in PDLs that were characterized by such OCLs. As shown in Figure 1, in bone areas in mice without PDLs, sclerostin expression and dendritic processes of OCys in *MVNP* mice were significantly reduced compared with WT mice. Sclerostin expression and dendritic processes of OCys were further reduced in *MVNP* mice at sites of PDLs (Figure 2A), which also had lower numbers of sclerostin-expressing OCys per bone area and decreased canalicular length compared with *MVNP* mice without PDLs or WT mice (Figure 2, B and C). These results suggest that OCys may contribute to the increased pagetic OCL formation and to formation of PDLs.

### Characteristics of primary OCys from bones of WT and MVNP mice.

**Figure 3:**
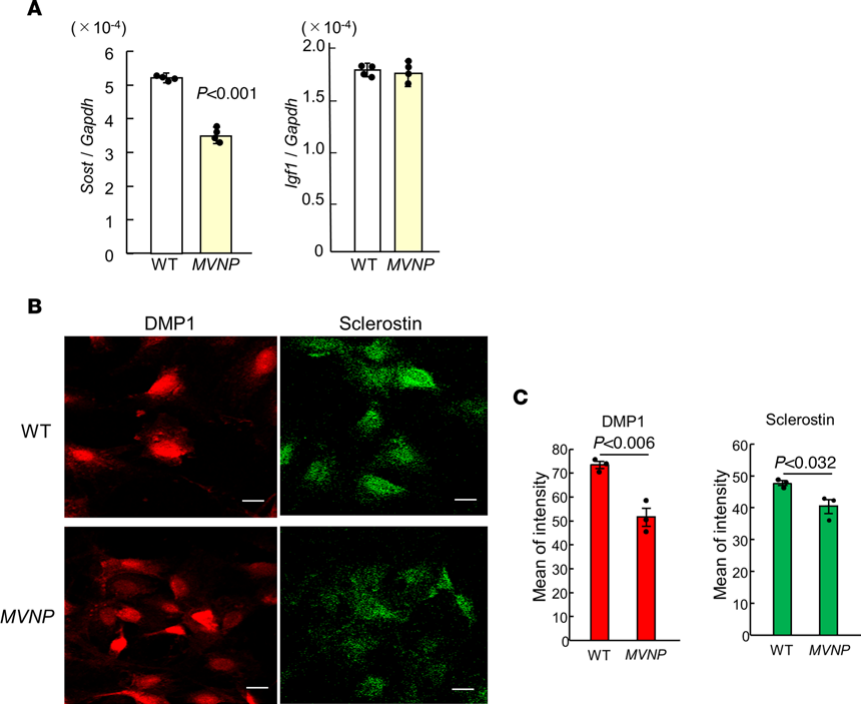
Characterization of primary OCys from WT and *MVNP* mice. (**A**) *Sost* and *Igf1* mRNAs: Primary OCys were isolated form long bones from 20-month-old WT and *MVNP* mice by sequential digestion with collagenase as described in Methods. Fractions 6–9 were used. RNA was extracted from 2 × 10^6^ cells from 4 individual WT or *MVNP* mice and analyzed by TaqMan PCR. Data for *Sost* and *Igf1* are the mean ± SEM (3 technical replicates from the mice) analyzed using the Mann-Whitney *U* test in **A** and **C**. (**B**) Sclerostin and DMPI protein: OCys were fixed, stained with anti-sclerostin or -DMP1 antibody and fluorescent secondary antibody conjugates, and examined by immunofluorescence. Scale bars: 10 μm. (**C**) The pixel intensity of positive cells was measured using a laser confocal microscope. Results shown relative pixel intensity of cells in 30 random cells from 3 wells, mean ± SEM from 1 average value per sample and analyzed using a 1-way ANOVA with Tukey’s test. The experiment was performed 3 times using different biological replicates with similar results.

Since sclerostin expression and dendrite formation are characteristics of mature OCys (11), and OCys produce IGF1 (12), we analyzed *Sost* and *Igf1* mRNAs in primary OCys isolated by collagenase digestion of long bones from 20-month-old WT and *MVNP* mice. *Sost* gene expression in OCys of *MVNP* mice was reduced by 30% compared with WT mice, while *Igf1* gene expression in OCys from WT and *MVNP* mice was unchanged (Figure 3A). Fluorescent immunostaining of primary OCys from *MVNP* mice showed decreased average intensity of staining for the OCy maturation markers DMP1 and sclerostin compared with OCys in WT mice (Figure 3, B and C). These results suggest that OCys from *MVNP* mice exhibit impaired maturation compared with WT OCys.

Since only a limited numbers of OCys can be obtained by collagenase digestion, we isolated OBs and OCys derived from bone outgrowth cells of WT and *MVNP* mice. Differentiated OBs can be detected in outgrowth cells in bones from *MVNP* and WT mice at day 15 of culture, and when cultured for an additional 15 days, they differentiate into OCy-like cells. Outgrowth cells from bone reflect the phenotype of OCys in bone (9) and demonstrate that OCy maturation appears decreased in OCys-like cells from *MVNP* mice (Supplemental Figure 1, A–C; supplemental material available online with this article; https://doi.org/10.1172/jci.insight.159838DS1). Furthermore, OCy-like cells from bones of *MVNP* mice produced and secreted less sclerostin than OCy-like cells derived from the other 3 genotypes (Supplemental Figure 1D).

### OCys from MVNP mice express increased RANKL compared with WT, Igf1-cKO, and MVNP/Igf1-cKO mice.

**Figure 4:**
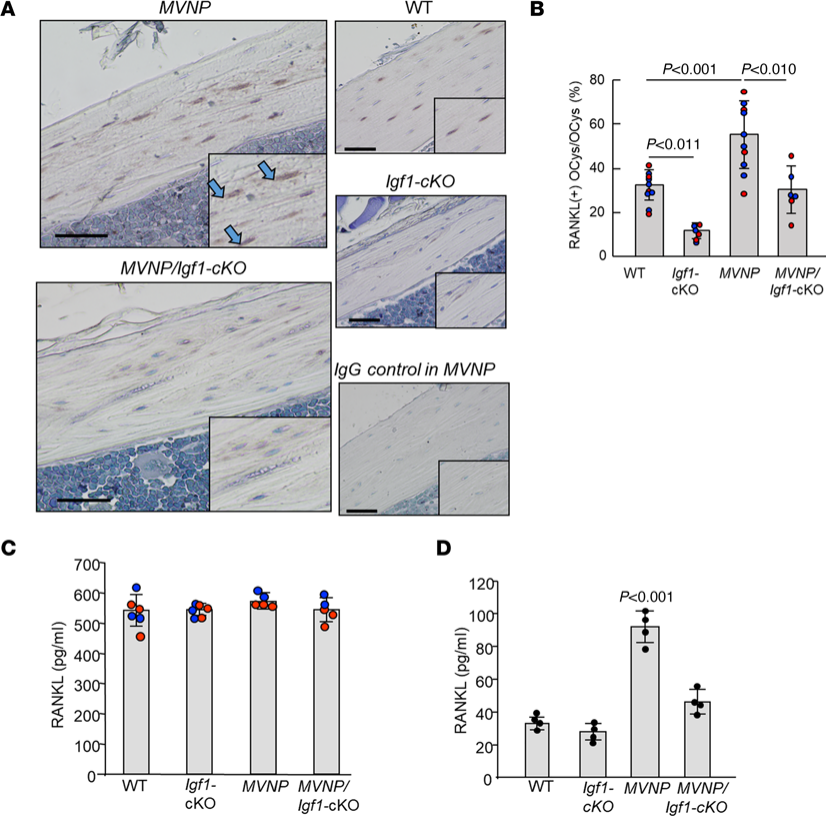
RANKL-positive OCys in 20-month-old WT, *Igf1*-cKO, *MVNP*, and *MVNP*/*Igf1*-cKO mice. (**A**) RANKL in OCys in decalcified femoral sections was immunostained as described in Methods. Scale bars: 50 μm. (**B**) The ratio of RANKL-positive cells/0.5 mm^2^ area was determined in bones from 10 WT, 6 *Igf1*-cKO, 10 *MVNP*, and 6 *MVNP*/*Igf1*-cKO mice. Blue dots are data from males and red from females, expressed as the mean ± SEM, analyzed using a 1-way ANOVA with Tukey’s test. (**C**) Serum RANKL was measured by mouse ELISA kit and expressed as mean ± SEM for 6 WT, 6 *Igf1*-cKO, 5 *MVNP*, and 5 *MVNP*/*Igf1*-cKO mice. No statistical difference was found between results from male (blue) and female (red) samples. (**D**) RANKL media conditioned by OCy-like cells (1 × 10^5^ cells/mL) from 30-day bone outgrowth cultures grown for 72 hours was analyzed by ELISA. Results shown are mean ± SEM and were analyzed by 1-way ANOVA with Tukey’s test.

Since OCy-derived RANKL plays a key role in OCL formation (10–15), we examined RANKL expression by OCys in bone sections of 20-month-old mice. We found that OCys of *MVNP* mice expressed increased RANKL compared with OCys of WT and *MVNP*/*Igf1*-cKO mice (Figure 4A). Interestingly, the numbers of RANKL-expressing OCys per bone area in *MVNP* mice were higher than in the other genotypes (Figure 4B). RANKL released into conditioned media (CM) of OCys from *MVNP* mice was also significantly higher than in OCy CM of the other genotypes (*P* < 0.01) (Figure 4D).

We then determined whether RANKL mRNA in primary OCys isolated by collagenase digestion was also increased in *MVNP* mice compared with WT mice and found this to be the case (*P* < 0.01) (Supplemental Figure 2, A and B). Thus, RANKL production by the 30-day outgrowth cells from bone reflected differences in RANKL production by primary OCys in WT and *MVNP* mouse bones, with increased RANKL production by *MVNP* OCy–like cells compared with WT OCy–like cells. Furthermore, RANKL was also increased in OCy-like cells from *MVNP* mice at the protein level (31-kDa band), which is the membrane-bound form of RANKL (Supplemental Figure 2, C–E).

### OCL-IGF1 increases RANKL production by OCys and induces formation of PD OCLs.

**Figure 5:**
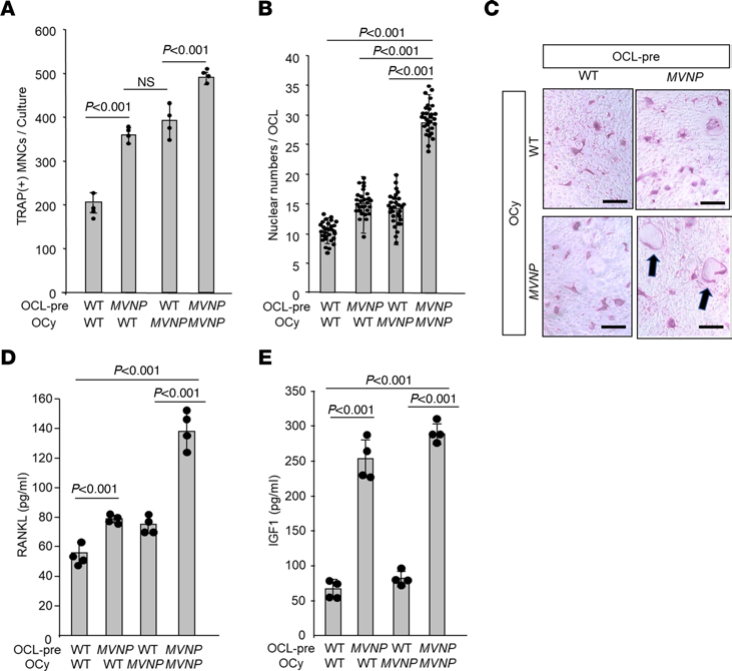
OCL formation by coculture with OCy-like cells from *MVNP* and WT mice. (**A**) OCL precursors from 16-month-old male WT and *MVNP* mice were cocultured with OCy-like cells from 15-month-old male WT and *MVN*P mice for 72 hours and stained for TRAP. Results are mean ± SEM (*n* = 4). Data for **A**–**E** were analyzed using 1-way ANOVA with Tukey’s test. (**B**) Nuclear numbers per OCL, expressed as the mean ± SEM of 30 randomly counted OCLs from each coculture. (**C**) OCL morphology from photomicrographs. Scale bars: 100 μm. Arrows point to large, polynucleated OCLs. (**D**) Media were collected at the end of the cultures shown in **A**, and RANKL protein was measured by ELISA and shown as mean ± SEM (*n* = 4). Biological replicates from female mice gave similar results (not shown). (**E**) The conditioned media in **D** were also assessed by IGF1 ELISA. Similar results were found in 2 biological replicates and with cells from female mice (not shown).

We then asked whether the large amounts of OCL-IGF1 secreted by *MVNP* mice could increase RANKL production by OCys and contribute to pagetic OCL formation in cocultures of OCL precursors and OCys from either WT or *MVNP* mice. When *MVNP* OCL precursors were cocultured with *MVNP* OCy–like cells, OCL formation and RANKL in culture media were increased 2.5-fold compared with cocultures of WT OCL precursors with WT OCy–like cells, with intermediate levels of OCL formation and RANKL production seen in WT/*MVNP* mixed cocultures (Figure 5, A, B, and D). As shown by the arrows in Figure 5C, PD-like hypermultinucleated OCLs only formed in cocultures of *MVNP* OCL precursors with *MVNP* OCy–like cells. IGF1 levels were only increased in media of cocultures containing OCL precursors from *MVNP* mice, regardless of whether OCys of WT or *MVNP* mice were in the coculture (Figure 5E). Thus, increased IGF1 is secreted by OCL precursors and OCLs from *MVNP* mice rather than by OCys in the cocultures.

Since IGF1 from OCLs could increase OCL formation via autocrine actions on OCLs (9), we examined the contribution of OCL-IGF1 to OCL formation in cultures of purified OCL precursors treated with an anti-IGF1 or anti–IGF1 receptor (anti-IGF1R) antibody in the absence of OCys. Treatment with anti–IGF1 or anti-IGF1R inhibited OCL formation by 25% in WT OCL precursor cultures and 40%–60% in *MVNP* OCL precursor cultures (Supplemental Figure 3A). We then assessed RANKL produced by OBs and OCys from *MVNP* and WT mice in CM of OBs and OCy-like cells cultured for 72 hours. Although both OBs and OCy-like cells secreted RANKL, OCy-like cells secreted more RANKL than OB (*P* < 0.001), and RANKL secretion was significantly higher in CM from *MVNP* OCys compared with CM of WT OCys (Supplemental Figure 3B). Thus, OCL-IGF1 from *MVNP* mice increases OCL formation in part via autocrine mechanisms, and via *MVNP* OCy–like cells, which express higher RANKL, and further increase OCL numbers and PD-like OCL formation (Figure 5, A–C).

### OCL formation in cocultures of OCL precursors and OCy-like cells is decreased by transducing OCys with Igf1r siRNA or treatment with anti-IGF1 or anti-IGF1R.

**Figure 6:**
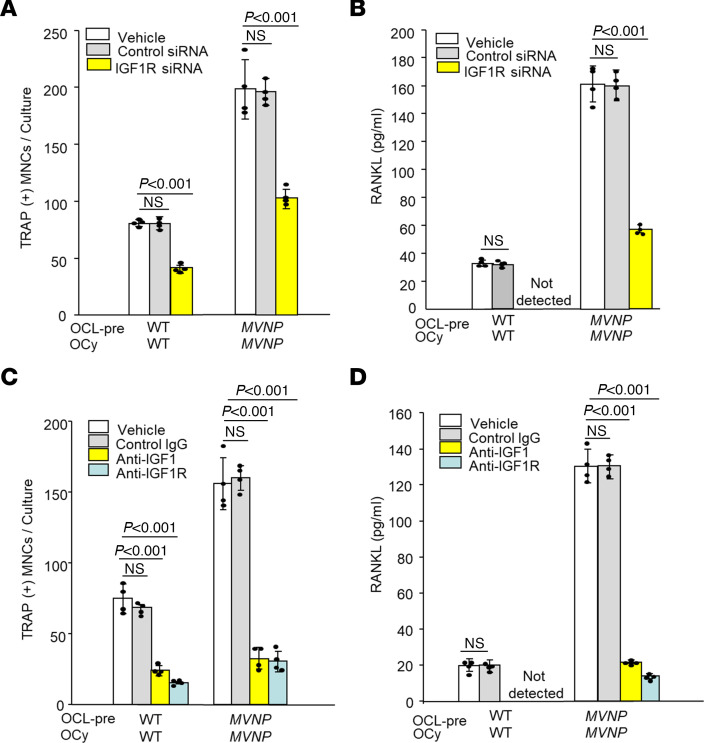
Role of IGF1 and receptor in OCL formation in cocultures from *MVNP* and WT mice. OCL precursors from 17-month-old mice and OCy-like cells from 15-month-old ones were cocultured for 72 hours as in Figure 5. (**A**) Numbers of TRAP-stained OCLs when OCy-like cells were transduced with IGF1R or control siRNAs prior to coculture. (**B**) RANKL protein in coculture conditioned media (CM) measured by ELISA. Results are with cells from male mice. Female mice gave similar results (not shown). (**C**) OCL formation in coculture treated with control rabbit IgG (20 ng/mL), anti-IGF1 (10 ng/mL), or anti-IGF1R (0.5 μg/mL). Cells were stained for TRAP. (**D**) RANKL protein in the CM from **C** measured by ELISA. (**C** and **D**) were repeated and performed with cells from female mice, with similar results (not shown).

We then asked whether OCL-IGF1 was directly increasing RANKL production in OCys. When *MVNP* OCys transduced with *Igf1r* siRNA (*MVNP*-IGF1R siRNA-OCy) or control siRNA were cocultured with *MVNP* OCL precursors, OCL formation and RANKL in CM of cocultures containing *MVNP*-IGF1R siRNA-OCys were significantly lower (by 65%) compared with *MVNP*-control siRNA-OCys cocultures (Figure 6, A and B). The *Igf1r* siRNA–transduced OCy-like cells from WT and *MVNP* mice still had suppressed IGF1R expression after 48 hours (Supplemental Figure 4).

Further, addition of anti-IGF1 or anti-IGF1R to OCL/OCy cocultures inhibited OCL formation and decreased RANKL levels in CM of *MVNP* OCL precursors cocultured with *MVNP* OCys (by 80%) compared with vehicle or control IgG cocultures (Figure 6, C and D). In addition, development of PD-like OCLs by *MVNP* OCL precursors was also decreased in these cocultures (data not shown). Similar inhibition of OCL formation was observed in coculture of WT OCL precursors and WT OCy (70%), but RANKL levels in CM were below the detection range of the RANKL ELISA. We also found that OCys in coculture adopted a spindle shape when the action of IGF1 was blocked.

### Bone resorption capacity of OCLs cocultured with OCy-like cells.

**Figure 7:**
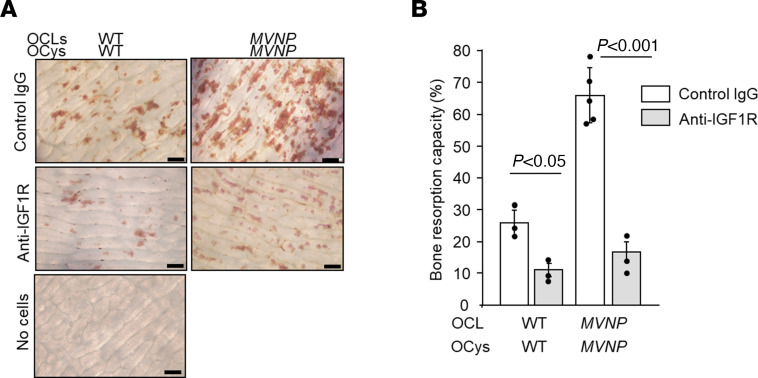
Bone resorption by OCLs formed in cocultures with OCy-like cells and role of IGF1. Mature OCLs (from 16-month-old female WT and *MVNP* mice) and OCy-like cells (from 19-month-old female WT and *MVNP* mice) were cocultured on bone slices with rabbit IgG (20 ng/mL), or anti-IGF1R (0.5 μg/mL) for 72 hours in 96-well plates. (**A**) Bone slices were stained with hematoxylin after removal of cells. Scale bars: 200 μm. Photomicrograph images are representative of 2 independent experiments using 2 biological replicates. (**B**) Bone resorption was assayed, and independent replicate values in each experimental group of bone resorption were plotted on the graph. The results are expressed as mean ± SEM, analyzed using a 1-way ANOVA with Tukey’s test. Each stained bone slice was divided into quadrants. Under the microscope, each quadrant was scored as + or – for pits in 500 squares using a 20 × 25 grid. Results are expressed as percentage of the 500 squares per slice scored positive for resorption. Similar results were seen for 2 independent biological replicates.

To test the bone resorption capacity of OCLs formed in cocultures of OCL precursors with OCys, the same numbers of OCLs and OCy-like cells were cocultured without RANKL on bone slices for 72 hours. Coculture of *MVNP* OCLs with *MVNP* OCy–like cells formed numerous large resorption pits, while coculture of WT OCLs with WT OCy–like cells formed low numbers of small pits (Figure 7A). Anti-IGF1R treatment of *MVNP* OCL precursors cocultured with *MVNP* OCys on bone slices decreased the bone resorption rate by 80% compared with bone resorption in control-IgG-treated cocultures (Figure 7B). In contrast, anti-IGF1R decreased bone resorption rates in cocultures of WT OCLs with WT OCy–like cells by 30%. WT OCy/*MVNP* OCL and *MVNP* OCy/WT OCL cocultures were not evaluated in the bone resorption pit analysis, because the number of OCLs formed and the concentration of RANKL in the coculture medium (Figures 5 and 6) were too low to demonstrate bone resorption.

### T-Igf1 mice expressed PDLs like those seen in MVNP mice.

**Figure 8:**
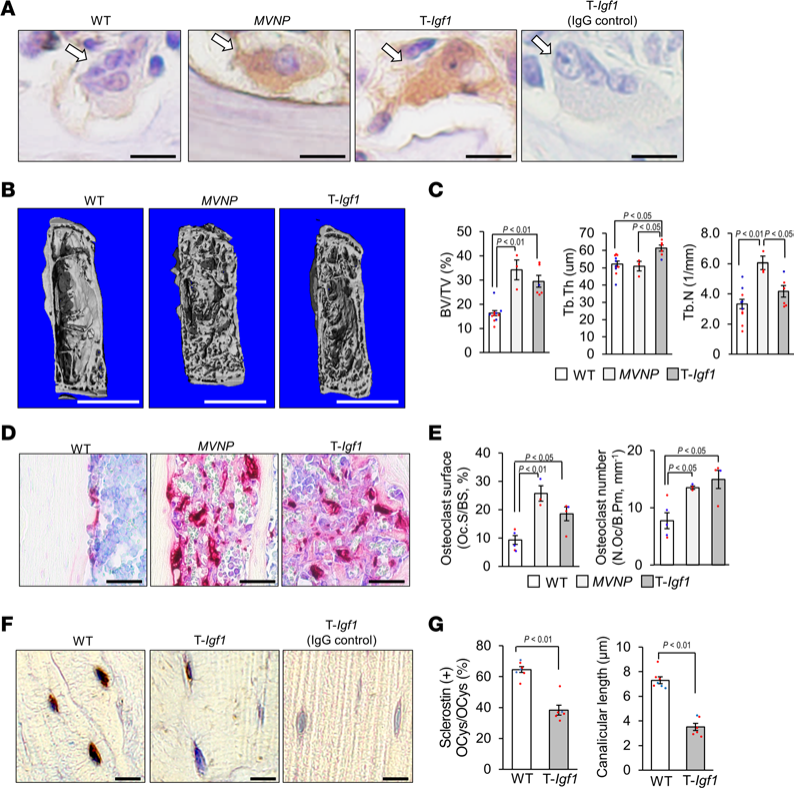
The detection of IGF1, bone structure, and histomorphometric analysis in WT and T-*Igf1* mice. (**A**) Detection of IGF1. Femur sections were stained with anti-IGF1. Arrows point to OCLs. Scale bars: 10 μm. Results are representative of 3 biological replicates. (**B**) Representative μCT images of fifth lumbar vertebrae. Scale bars: 1.0 mm. (**C**) Bone volume and structural parameters of trabecular bone in vertebrae. Results are mean ± SEM for WT, with blue indicating males and red females (5 males, 6 females, 16 ± 3 months), *MVNP* with PDL (3 females, 19 ± 1 months), and T-*Igf1* with PDL (1 male, 5 females, 15 ± 1 months). The data were analyzed using 1-way ANOVA with Tukey’s test. (**D**) OCL morphology. OCLs in vertebral sections were stained for TRAP. Scale bars: 50 μm. Staining results are representative of 3 biological replicates. (**E**) Bone morphometric analysis. Results are expressed as the mean ± SEM for WT (3 males, 2 females, 16 ± 3 months), *MVNP* with PDL (1 male, 2 females, 21 ± 2 months), and T-*Igf1* with PDL (1 male, 3 females, 15 ± 1 months). The data were analyzed using 1-way ANOVA with Tukey’s test. (**F**) Sclerostin in OCys. Femur sections from these mice were stained with anti-sclerostin antibody as described in Methods. Scale bars: 10 μm. Staining results are representative of 3 biological replicates. (**G**) Ratio of sclerostin-positive OCys/total OCys per area and canalicular length (0.25 mm square). Results are expressed as the mean ± SEM from WT (each 3 males and 4 females, 15 ± 1 months) and T-*Igf1* with PDL (1 male, 4 females, 15 ± 1 months) from the same sections shown in **F** as described in Methods analyzed using 1-way ANOVA with Tukey’s test. BV/TV, trabecular bone volume fraction; Tb.Th, trabecular thickness; Tb.N, trabecular number; N.Oc/B.Pm, number of osteoclasts per bone perimeter.

To test whether high levels of OCL-IGF1 alone are sufficient to induce PDLs and pagetic OCLs, we generated a T-*Igf1* mouse that overexpresses IGF1 in OCLs. To confirm the role of OCL-IGF1 obtained in *MVNP* mice, we selected T-*Igf1* mice as founders with the same level of OCL-IGF1 as in *MVNP* mice (mice expressing twice as much IGF1 as in WT mice). T-*Igf1* mice were aged to assess whether they develop PDLs and pagetic OCLs at 16 months of age for comparison to similar ages of *MVNP* and WT mice. OCLs from bone marrow (BM) culture of these T-*Igf1* mice express IGF1 at the same level as *MVNP* mice and express twice as much IGF1 as WT, as shown in Supplemental Figure 7A. Interestingly, the expression of IL-6 in OCLs of T-*Igf1* mice was at the same level as in WT mice and lower than in *MVNP* mice. Furthermore, OCL-IGF1 levels in the bones of T-*Igf1* and *MVNP* mice were detected at similar levels and more strongly stained with anti-IGF1 than in WT, as shown in Figure 8A. There was no difference in plasma IGF1 levels between WT and T-*Igf1* mice (Supplemental Figure 7B).

T-*Igf1* mice developed the same PD phenotype as *MNVP* mice (Figure 8B). Mice of this age were examined because we most consistently find PDLs in *MVNP* mice at 12 months of age or older (9). PDLs were detectable in 50% of T-*Igf1* mice (6 of 12) by μCT and histological analysis (Table 1) and found in vertebrae, femurs, and tibiae. The number of PDLs formed ranged from 1 to 5 in each bone. The results of μCT analysis (Figure 8C) and histological analysis (Figure 8E) in T-*Igf1* mice were similar to *MVNP* mice. Very large OCLs also appeared in T-*Igf1* mice (Figure 8D). These results suggest that IGF1 is the major OCL product driving PDL formation in the *MVNP* model. Even in the absence of MVNP and IL-6 in OCLs, IGF1 alone facilitates PDL formation.

Furthermore, OCys in T-*Igf1* mice showed lower sclerostin staining compared with WT mice (Figure 8F), and the numbers of sclerostin-expressing OCys per bone area were also significantly reduced compared with WT mice (Figure 8G). Canalicular length of OCys in T-*Igf1* mice was also shorter in *MVMP* mice (Figure 8G). DMP1 expression was also lower in OCys of T-*Igf1* mice than in WT (data not shown). These results are similar to the observations in *MVNP* mice shown in Figure 1, A and B.

### Detection of p-Akt, RANKL, and p16^INK4A^ in WT, MVNP, and T-Igf1 mice.

**Figure 9:**
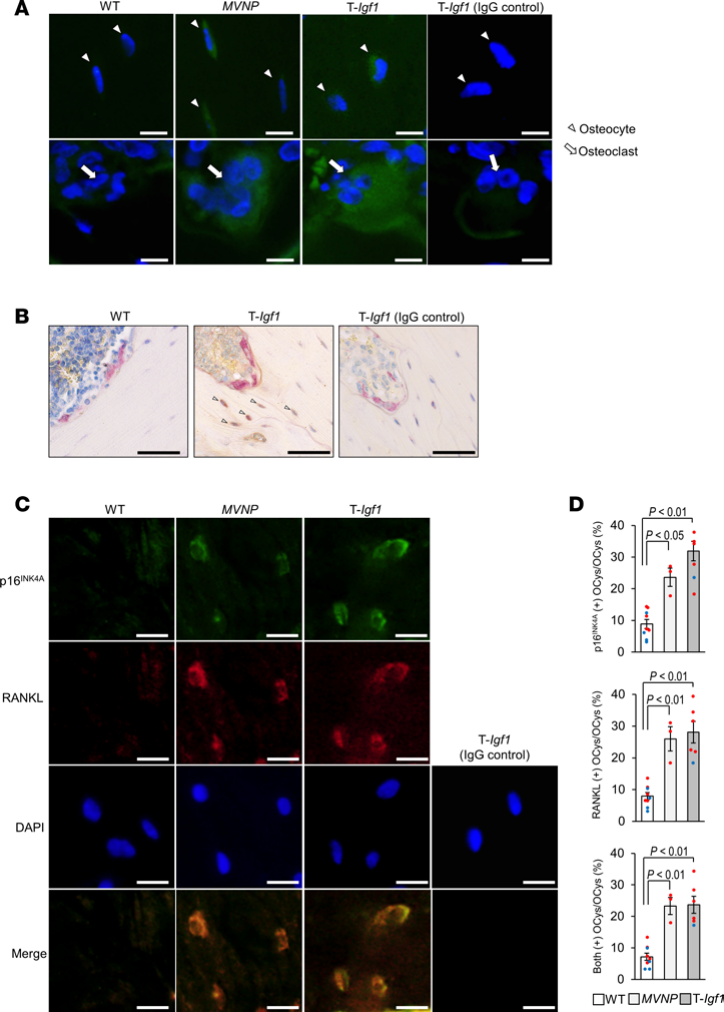
Detection of p-Akt, RANKL, and p16^INK4A^ in WT, *MVNP*, and T-*Igf1* mice. (**A**) Detection of p-Akt. Femur sections were stained with an anti–p-Akt antibody. Scale bars: 10 μm. Staining results are representative of 3 biological replicates. (**B**) RANKL in OCys. Femur sections were stained with an anti-RANKL antibody and for TRAP as described in Methods. Scale bars: 50 μm. Staining results are representative of 3 biological replicates. (**C**) RANKL and p16^INK4A^ in OCys. Sections stained with anti-p16^INK4A^ and anti-RANKL. (**D**) The ratio of RANKL- and p16^INK4A^-positive cells/250 μm^2^ at 500 μm below the growth plate was determined in bones of WT (4 males, 5 females, 16 ± 3 months), *MVNP* (3 female, 19 ± 1 months) and T-*Igf1* (1 male, 5 female, 15 ± 1 months). Blue circles are data from males and red from females, expressed as the mean ± SEM, analyzed using 1-way ANOVA with Tukey’s test.

Since IGF1 stimulates phosphorylation of PI3K/Akt via IGF1R (9), we examined p-Akt expression in bones from these mice. Interestingly, more p-Akt staining was observed in T-*Igf1* and *MVNP* than in WT mice (Figure 9A). OCys and OCLs from *MVNP* and T-*Igf1* mice stained positively for p-Akt compared with OCys and OCLs from WT mice. These results suggest that OCL-IGF1 not only transduces signals to OBL and OCys via IGF1R, but also has autocrine effects on OCLs, as shown Supplemental Figure 3A.

Next, we assessed IGF1 effects on OCys by histomorphology. We stained for TRAP and RANKL by IHC of bones from T-*Igf1* and WT mice. OCys near OCLs expressed more RANKL in T-*Igf1* mice than in WT mice (Figure 9B). These results are similar to RANKL production in OCys of *MVNP* mice, as shown Figure 4.

Farr et al. recently reported that the removal of senescent OCys reduced RANKL production by OCys and restored osteogenesis (16). Therefore, we examined the production of p16^INK4a^ and RANKL in *MVNP*, T-*Igf1*, and WT mice. p16^INK4a^ and RANKL were visualized by immunofluorescent staining in single cells. The expression of p16^INK4a^ and RANKL in *MVNP* and T-*Igf1* mice was stronger than in WT (Figure 9C). p16^INK4a^- and RANKL-positive OCys were counted within 250-μm^2^ areas at 500 μm below the growth plate. The ratio of p16^INK4a^- and RANKL-positive OCys of *MVNP* and T-*Igf1* mice was increased 2.5- to 3.5-fold compared with WT. Moreover, p16^INK4a^/RANKL double-positive OCys accounted for 25% of total OCys in *MVNP* and T*-Igf1* mice, which was 3-fold higher than WT (Figure 9D). The results confirm previous observations that RANKL production is part of senescence-associated secretory phenotype of OCys.

## Discussion

In the current study, we found that OCys in PD were both functionally and morphologically abnormal. As shown in Figure 1, OCys adjacent to PDLs of a PD patient and from *MVNP* mice have shorter dendritic processes compared with OCys from a normal patient and WT mice respectively. *MVNP* OCys also expressed lower levels of genes associated with OCy maturation, such as sclerostin, DMP1 and FGF23, and higher levels of collagen type1 and BSP compared with *Igf1*-cKO, *MVNP/Igf1*-cKO, and WT mice (Supplemental Figure 1). These results suggest that OCy maturation may be impaired or delayed in PD. In support of this notion are our similar findings for expression of OCy maturation markers in primary osteocytic cells isolated from *MVNP* and WT mouse bones (Figure 3).

We recently reported that pagetic OCLs and OCLs expressing *MVNP* produce high levels of IGF1 that are required for development of pagetic OCLs and PDLs in *MVNP* mice (8, 9). Our current results suggest that these changes in OCy marker gene expression may reflect effects of OCL-IGF1 on OCy differentiation. PDLs were also seen in T-*Igf1* mice created to study the contribution of OCL-IGF1 to OCy (Figures 8 and 9). This seems likely, since OCys from *MVNP/Igf1*-cKO mice have more normal-appearing OCys. Further, IGF1 treatment of WT or *MVNP* OCys decreased sclerostin expression (Supplemental Figure 5). The shorter dendritic processes of OCys in the bone biopsy of a PD patient and *MVNP* mice could reflect a block in transition of osteoid OCys to mature OCys or decreased expression of genes involved in dendrite formation, such as or E11 or kalirin (17).

Interestingly, OCys in *MVNP* mice with PDLs had significantly decreased canalicular length and sclerostin expression per bone area compared with OCys in *MVNP* mice lacking PDLs (Figure 2). This difference may be due to the increased OCL numbers/bone area in bones with a PDL, which could result in increased local OCL-IGF1 levels compared with bones from *MVNP* mice without PDLs. Our finding that serum levels of IGF1 (9) and sclerostin in the 4 genotypes were similar (Figure 1D), in combination with the presence of normal appearing OCys in *MVNP/Igf1-*cKO mice, support the importance of high local IGF1 in PDL formation.

The basis for impaired differentiation of OCys in *MVNP* mice is unclear. Possibly, this may result from increased expression of Runx2 in late OBs induced by OCL-IGF1. Komori et al. reported that forced expression of Runx2 in late-stage OBs suppresses their differentiation into OCys, using a mouse model in which Runx2 was forcibly expressed in late-stage OBs driven by an osteocalcin promoter (18, 19). We previously found that IGF1 increases the expression of EphB4 and Runx2 in primary OBs from *MVNP* mice (8). As shown in Supplemental Figure 6A, the expression of Runx2 was only increased in *MVNP* OCys derived from day 30 cultures. Furthermore, when IGF1 was added to cultures of late OBs (day 15 of culture), Runx2 expression in OBs in *MVNP* mice was increased 6.9-fold compared with WT mice treated with vehicle (Supplemental Figure 6B). These results suggested that OCL-derived IGF1 induces the expression of Runx2 in OBs and impedes their differentiation into OCys.

In support of this notion, primary OCys isolated from *MVNP* and WT mouse bones showed similar changes in OCy gene expression as OCy-like cells present in 30-day outgrowth cells from bones of *MVNP* and WT mice. *Sost* mRNA levels in primary OCys from *MVNP* mice were 30% lower than those in primary OCys from WT mice. Sclerostin expression by immunohistochemical analysis of primary OCys showed a similar pattern of results (Figure 3).

OCys present in the canalicular lacunae of *MVNP* mice showed increased RANKL expression and secretion compared with *MVNP/Igf1*-cKO, *Igf1*-cKO, and WT mice (Figure 4A). The numbers and percentages of RANKL-expressing OCys were significantly increased in *MVNP* mice compared with the other genotypes (Figure 4B), although serum RANKL was similar in all genotypes. Further, RANKL in CM of OCys from *MVNP* mice was significantly higher than in CM of the other genotypes (Figure 4, C and D). These results suggest that RANKL production by OCys is increased in PD patients who have prolonged exposure to high local OCL-IGF1. These high local levels of RANKL may induce formation of large numbers of PD OCLs that secrete high levels of IGF1. Since PD OCL precursors are hyperresponsive to RANKL (20, 21), IGF1 may then induce OCy RANKL production that further increases PD OCL formation, eventually resulting in a PDL. Consistent with this notion, coculture of *MVNP* OCL precursors and *MVNP* OCys showed increased pagetic OCL formation, and the CM of these cocultures contained higher RANKL levels compared with cocultures of WT OCL precursors with OCys from WT and *MVNP* mice (Figure 5). Importantly, when *MVNP* OCys transduced with *Igf1r* siRNA (*MVNP*-*IGF1R* siRNA-OCy) or control siRNA were cocultured with *MVNP* OCL precursors, OCL formation and RANKL levels in CM of *MVNP* OCL precursors with *MVNP*-*Igf1r* siRNA-OCys were significantly lower (65%) versus *MVNP*-control siRNA-OCys cocultures. Further, addition of anti-IGF1 or anti-IGF1R antibodies to these cocultures similarly suppressed expression of a pagetic phenotype in *MVNP* OCLs. In addition, anti-IGF1R treatment of *MVNP* OCL precursors cocultured with *MVNP* OCys on bone slices decreased the bone resorption rate by 80% compared with control-IgG-treated cocultures. In addition, the observation of p-Akt in OCys of bone sections from *MVNP* and T-*Igf1* mice suggests that IGF1 activates signaling in OCys (Figure 9A). These results demonstrate that PD OCL-IGF1 induces RANKL and decreases sclerostin expression in OCys via IGF1R on OCys.

Pagetic OCLs in *MVNP* mice express elevated IGF1, and OCL-cKO of *Igf1* blocks local PDL formation in this model, while further increases in IGF1 secretion by OCLs may increase PDLs. We showed previously that IL-6, while insufficient to initiate pagetic lesions, could enhance the effects of IGF1 (8). To evaluate whether elevated levels of OCL-IGF1 alone are sufficient to induce PDL and pagetic OCLs, we generated a T-*Igf1* mouse that overexpresses IGF1 in OCLs. As shown in Figure 8, PDLs were induced by overexpression of IGF1 in OCLs. At 16 months of age, PDLs were found in 54% of T-*Igf1* mice, allowing us to distinguish the contribution of IGF1 effects on OCys in PD, and the increased OCL-IGF1 secretion by PDLs in the absence of *MVNP* expression was sufficient to induce PD.

Finally, 25% of OCys at sites of PDLs showed senescence, and RANKL secretion was observed in these OCys, suggesting a relationship between senescence and PDLs (Figure 9D). Most recently, Farr et al. reported that deletion of p16 from OCys reduced RANKL from OCys (16). Insulin/IGF1 signaling induces intracellular oxidative burden and associated oxidative damage (22). IGF1 induces specific p53 acetylation via inhibition of SIRT1, leading to premature senescence (23).

Taken together, our results with T-*Igf1* and *MVNP*/*Igf1*-cKO mice support a model in which OCL-IGF1 induces PD OCL formation and PDLs. A small collection of PD OCLs secrete high local levels of IGF1 that induce local OCy RANKL production, suppress OCy sclerostin expression, and enhance formation of PD OCLs from OCL precursors that are hyperresponsive to RANKL. This increases local bone destruction. OCy-derived RANKL could in turn recruit additional PD OCL precursors to this specific site in bone, analogous to OCL recruitment by apoptotic OCys. This could result in multiple cycles of PD OCL formation that increases local bone destruction and can induce local rapid bone formation via the expression of the coupling factors EphB2 on OCLs and EphB4 on OBs (8). IGF1 further stimulates local bone formation and development of PDLs (8, 9) in a bone site with low sclerostin levels. Thus, our results suggest that OCys play a key role in PDL formation in PD.

## Methods

### Chemicals.

Rabbit IgG (catalog AB-105-C), anti-IGF1 (catalog AF791), and anti-IGF1R (catalog MBA391-100) were purchased from R&D Systems. αMEM was from Thermo Fisher Scientific and FBS from Sigma-Aldrich. An anti–p-Akt antibody (catalog 9271) was purchased from Cell Signaling Technology. An anti-p16^INK4A^ antibody (catalog 03119) was purchased from GeneTex.

### Animal studies.

Animals were housed at Virginia Commonwealth University in individually ventilated cages in a barrier vivarium, which excludes all known mouse viruses and parasites and most bacteria (including *Helicobacter*). The mice were fed standard mouse chow (irradiated Teklad LM-485 diet) and autoclaved water. Mice of both sexes and multiple ages were euthanized under isoflurane anesthesia, followed by cervical dislocation for collection of bone tissues, which were shipped overnight to Indiana University School of Medicine in DMEM plus10% FBS with penicillin/streptomycin at 20°C.

### Generation of mice with cKO of Igf1 in OCLs.

Mice with cKO of *Igf1* in OCLs were generated by breeding *Igf1^fl/fl^* mice that carry *loxP* sequences flanking exon 4 of the gene (24) (Jackson Laboratory, stock 016831) with transgenic mice expressing Cre recombinase under the control of a 2.3-kb murine *TRAP* promoter (25) to generate *TRAP*-Cre (+)/*Igf1^fl/fl^* mice. These mice were further bred with WT or *TRAP-MVNP* mice (5) to generate mice of the following 4 genotypes: (a) WT, (b) *Igf1*-cKO, (c) *MVNP*, and (d) *MVNP/Igf1*-cKO, as we previously reported and characterized (9). All mice were on a C57BL/6J background. At each generation, only 1 parent carried the *TRAP*-*MVNP* transgene and only 1 carried the *TRAP*-Cre transgene, so offspring carrying either were heterozygous for the transgene. Since our previous studies showed that 40% of *MVNP* mice at 16–22 month of ages (average 20 months old) had a detectable PDL, we used mice in this age range for this study. All experiments were performed using mice of both sexes. In previous studies, μCT and histomorphometry showed no statistically significant differences in *MVNP*-induced bone changes between male and female mice (9).

### Generation of mice with Igf1 in OCLs.

We expressed the murine IGF1 isoform containing the E peptide (isoform 4), which increases local bioavailability of IGF1 while minimizing release into the systemic circulation (26). The founder mice were generated and bred with WT mice to obtain germline transmission. OCLs were isolated from 1 transgenic offspring and 1 WT control from founder mice under the control of a 2.3-kb murine *TRAP* promoter (22). IGF1 expression was assessed by Western blot analysis. The mice are on a C57BL/6J background. We identified the line whose OCL-IGF1 expression level most closely matches that of *MVNP* mice, using the same approach that examined whether overexpression of IL-6 in OCLs was sufficient to induce PD/PDLs (27). The mice were aged to assess wheher they develop PDLs and pagetic OCLs and OCys at 16 months of age in comparison with 12- and 18-month-old *MVNP* and WT mice.

### PD patient and healthy donor.

Deidentified resin-embedded bone sections from a transiliac crest bone biopsy of a 58-year-old female patient with PD and from a 30-year-old female patient as a healthy control were provided by Brendan Boyce (University of Rochester, Rochester, New York, USA). Both individuals had been treated with calcein and tetracycline before biopsy. The samples were embedded without decalcification in methyl methacrylate. Sections were stained with an anti-sclerostin antibody (Abcam, catalog ab63097). Histological examination of bone sections from this PD patient and healthy donor were previously reported (9).

### Immunohistochemical analysis.

The femurs from 20-month-old mice were fixed in 10% buffered formalin and decalcified in 10% EDTA for 2 weeks at 4°C and embedded in paraffin. Longitudinal sections (5 μm) were cut and mounted on glass slides. Deparaffinized sections were treated with 1% horse serum for 1 hour, followed by addition of primary antibodies against sclerostin (Abcam, catalog ab63097), RANKL (Santa Cruz, catalog sc-59982), or control rabbit IgG (Santa Cruz, catalog sc-2027). The sections were incubated overnight and then stained with anti–rabbit IgG conjugated to horseradish peroxidase (Vector Laboratories). Scoring of staining intensity was performed by a blinded observer, using a scale of 1–4+ to grade the staining intensity.

For immunofluorescence, 4 × 10^3^ isolated OCys or OCy-like cells were cultured with 10% FCS in αMEM overnight on 4-well chamber slides (Falcon, 354104) overnight and fixed with 4% paraformaldehyde for 30 minutes at room temperature. Cells were permeabilized in 0.1% Triton X-100 for 5 minutes before staining. Goat anti-DMP1 (Abcam, catalog ab81985) or rabbit anti-sclerostin (Abcam, catalog ab63097) was added for 12 hours, with goat or rabbit IgG (R&D Systems) as staining control. Anti-goat Alexa Fluor 594 conjugate (Life Technologies, catalog A11058) or anti-rabbit Alexa Fluor 488 conjugate (Invitrogen, catalog 11008) was then added for 2 hours. Sections were examined by confocal (Zeiss 710) and fluorescence microscopy (Olympus IX73).

Cell morphological studies employed fluorescent staining of F-actin filaments with Alexa Fluor 488–phalloidin conjugate to visualize OCy dendritic processes (Thermo Fisher Scientific, catalog A12379) and were examined by fluorescence microscopy.

### Measurements of canalicular lengths and staining for sclerostin and RANKL.

Lumbar vertebrae were decalcified in 10% EDTA at 4°C for 2 weeks and embedded in paraffin. The decalcified sections were immunostained for sclerostin, and canalicular lengths were measured using ImageJ software (NIH). The count of positive OCys for each antibody was performed in a square at 500 μm below the growth plate.

### Isolation of primary OCys from mouse long bones.

Primary osteocytes were isolated from femurs and tibiae of WT and *MVNP* mice, according to a method previously described by Miyagawa et al., with modifications (28). Briefly, mouse tibiae and femurs were minced into 0.5-mm pieces and digested with 1.25 mg/mL collagenase (Wako) in Ca^2+^-free, Mg^2+^-free Hanks’ balanced salt solution (HBSS) at 37°C. Cells released after the first and second (15 minutes each) and third to fifth (20 minutes each) digestion were collected through a 100-μm nylon cell strainer as fractions 1 to 5, respectively. Residual bone pieces were treated with 4 mM EGTA in Ca^2+^-free, Mg^2+^-free HBSS for 15 minutes and then with 1.25 mg/mL collagenase for 20 minutes at 37°C to collect cells for OCy-rich fractions (fractions 6 to 9).

### Isolation of OBs and OCy-like cells.

After flushing the BM from tibiae and femurs of WT, *Igf1*-cKO, *MVNP,* and *MVNP/Igf1*-cKO mice, the bones were cultured in αMEM plus 10% FCS for 15 days. The original bone was removed, and the outgrowth cells from the bone were treated with 0.25% trypsin and 0.05% EDTA for 10 minutes at 37°C. These cells were used as primary OBs without further passage (8, 9). Cells were either stained for alkaline phosphatase or with alizarin red, or cell lysates were collected and analyzed for protein expression. Similarly, cells separated by trypsin from the outgrowth cells of bones that had been cultured for 30 days were used as OCys-like cells without further passage. As shown Supplemental Figure 1A, cells derived from day 15 outgrowth cells of bones expressed OB makers. Cells from day 30 culture expressed the OCy markers sclerostin, DMP1, and ORP150 by Western blotting and were used to determine RANKL production by OCys and for coculture with OCL precursors for assessing OCL formation.

### OCL formation from purified OCL precursors.

Nonadherent BM cells were harvested and enriched for CD11b^+^ mononuclear cells using CD11b microbeads (MACS, 120-000-300) and a Miltenyi Biotec MACS magnetic cell-sorting system. These cells were cultured with 10 ng/mL M-CSF (R&D Systems) in αMEM containing 10% FCS for 3 days. This stage of purified OCL precursor expresses RANK receptor and TRAP (8) and forms OCLs in the presence of 50 ng/mL RANKL for 2–4 days. The cells were stained for TRAP (Sigma-Aldrich), and TRAP^+^ multinucleated cells (≥3 nuclei/cell) were scored as OCLs.

### Coculture of purified OCL precursors and OCy-like cells.

OCL precursors (5 × 10^4^/well) and OCy-like cells (5 × 10^3^/well) isolated by the methods described above were cocultured in αMEM with 10% FCS for 72 hours in 96-well plates.

### Transfection of Igf1r siRNA into OCy-like cells.

OCy-like cells (5 × 10^3^) were plated in 96-well plates 12 hours before transfection with 100 nM siRNAs. Control siRNA (Cell Signaling, 6568) or mouse-specific *Igf1r* siRNA (Cell Signaling, 12482) were transfected into OCy-like cells as described previously (29).

### Isolation of mature OCLs from BM cultures.

BM cells flushed from long bones of WT or *MVNP* mice were cultured (2.5 × 10^7^ cells/10-cm dish) with 10 ng/mL M-CSF for 3 days, followed by 50 ng/mL RANKL for 4 days as described previously (8). At the end of culture, trypsin-EDTA (Corning) was added for 3 minutes to remove non-osteoclastic cells. OCLs were released from the plates by gently scraping with a rubber policeman.

### Coculture of OCLs and OCy-like cells for bone resorption.

OCLs (2 × 10^3^/well) and OCy-like cells (5 × 10^3^/well) were cocultured on bovine bone slices (Immunodiagnostic Systems, DT-1BON1000-96) in 96-well plates with αMEM plus 10% FCS with and without anti-IGF1R (0.5 μg/mL) for 72 hours. The cells were then removed, the bone slices stained with acid hematoxylin, and the areas of bone resorbed determined as previously described (30).

### RNA extraction and real-time PCR analysis.

Total RNA was extracted using TRIzol (Invitrogen), treated with DNase (Qiagen), and reverse transcribed with random hexamers (Promega) and SuperScript II (Invitrogen). cDNA was analyze using TaqMan with Real Time PCR (Applied Biosystems). To generate a standard curve for real-time PCR, amplicons of interest were first cloned into a pT7-blue vector (Novagen), and serial 10-fold dilutions of the plasmid included in the assay. The copy number of the target cDNA in each sample was estimated by referring to the standard curve, which was standardized to that of *Gapdh* in each sample. Specific primers were *Sost* forward, 5′-TCCTGAGAACAACCAGACCA-3′ and reverse, 5′-GCAGCTGTACTCGGACACATC-3′; *Igf1* forward, 5′-ACCGAGGGGCTTTTACTT CA-3′ and reverse, 5′-TGGCTCACCTTTCCTTCTCC-3′; *Tnfsf11* forward, 5′-AGCCATTTGCACACCTCAC-3′ and reverse, 5′-CGTGGTACCAAGAGGACAGAGT-3′; *Gapdh* forward, 5′-GTGTTCCTACCCCCAATGTG-3′ and reverse, 5′-ATAGGGCCTCTCTTGCTCAG-3′.

### Sclerostin, RANKL, and IGF1 ELISAs.

Collected mouse sera were stored at –80°C until tested. Sclerostin and IGF1 were measured using ELISA kits for murine Sost (Abcam, ab213889) and murine IGF1 (Abcam, ab100695), and RANKL was measured using an ELISA kit for murine/rat RANKL (R&D Systems, MTR00), according to the manufacturers’ instructions.

### μCT and histomorphometry.

Femora, tibiae, and vertebrae from WT, TRAP-*Igf1*, and *MVNP* mice at 16 months of age were fixed in 10% buffered formalin at 4°C. Bone microstructure analyses were performed using a μCT scanning system (Viva CT 40, Scanco Medical) with an isotropic voxel size of 10.5 μm and the scanner settings of 55 kVp, 25 μA, and 350 ms integration time. Structural parameters were analyzed in reconstructed 3-dimensional images using evaluation software (μCT v1.6, Scanco Medical) according to the recommended guideline (31). The regions of interest were defined using previously described methods (32, 33). The cancellous bone and marrow compartments of the L5 vertebral body were examined between the cranial and caudal growth plates. The cortical bones parameters were analyzed in 100 slices at the tibial midshaft, starting 5.5 mm from the proximal metaphysis. The μCT data were then exported as a sequence of 8-bit DICOM grayscale images, and simultaneous multiplanar reconstructed (MPR) images were viewed using ImageJ software.

The lumbar vertebrae were decalcified in 10% EDTA at 4°C and embedded in paraffin. OCLs containing active TRAP were stained red, as described by Liu et al. (34). OCL perimeter (OCL surface/bone surface, Oc.S/BS) was defined as the length of bone surface covered with TRAP^+^ multinuclear cells. OB perimeter (Ob.S/BS) was also measured in the same field.

### Statistics.

Significance was evaluated using 1-way ANOVA with Tukey’s test. Differences with *P* less than 0.05 were considered significant.

### Blinding.

To avoid bias, all data were collected in a blinded fashion, with the observer unaware of the experimental group. Key studies were performed by more than 1 individual to confirm observational consistency.

### Study approvals.

All animal studies were performed as described in approved IACUC protocols from Virginia Commonwealth University and Indiana University and an ACURO protocol from the Department of Defense, in accordance with the principles and procedures outlined in the NIH *Guide for the Care and Use of Laboratory Animals* (National Academies Press, 2011). Human patient samples used deidentified archival material not collected for this study and were IRB exempt.

## Author contributions

GDR, JMC, and NK designed the study, interpreted the data, and wrote the manuscript. KM, HT, PLM, JDC, and NK performed the experiments. MAS and JJW designed and generated the transgenic mice.

## Supplementary Material

Supplemental data

Supporting data values

## Figures and Tables

**Table 1:**
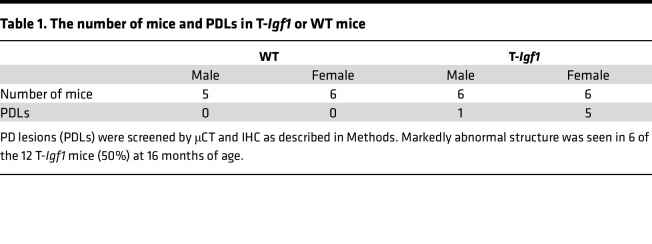
The number of mice and PDLs in T-*Igf1* or WT mice

## Version 1 06/20/2023

In-Press Preview

## Version 2 07/24/2023

Electronic publication
