# Supplementary material for: Osteoclast-derived IGF1 induces RANKL production in osteocytes and contributes to pagetic lesion formation
Source: JCI Insight. 2023 Jul 24;8(14):e159838. doi: 10.1172/jci.insight.159838 (PMC10443794; doi:10.1172/jci.insight.159838)
Supplement: Supplemental data [file jciinsight-8-159838-s027.pdf]

Supplemental Methods:

To distinguish osteocytic from osteoblastic cells (OBL), a series of marker genes were examined (Robling and Bonewald, 2020; Guo et al., 2010). OBL-selective ones were bone sialoprotein (BSP) and collagen 1a1 (Col-1a). OCy-selective ones were sclerostin (Sost), hypoxia-upregulated-1 (oxygen-regulated protein, ORP150), fibroblast growth factor 23 (FGF23) and dentinal matrix protein-1 (DMP-1). Osteocalcin is expressed in both late OBL and in OCys. Immunoblotting. Total proteins were extracted from OCLs, OBs, or OCys with RIPA buffer, and the cell lysates (10µg/lane) loaded onto Bio-Rad Mini-PROTEAN Precast Gels. The resolved proteins were transferred onto nitrocellulose membranes (TGX Membrane; Bio-Rad) using the Trans-Blot Turbo Transfer System (Bio-Rad). The membranes were stained with Ponceau S and cut into strips based on the molecular weight markers. Membranes were then exposed to primary antibodies overnight at 4°C and incubated with anti-IgG antibodies conjugated to horseradish peroxidase (HRP) for 1 hour. The blots were washed and specifically bound HRP was visualized by a Super Signal West Dura Extended Duration System (Cell Signaling). The following antibodies were used for detection of IGF1 (Abcam ab63926), IGF1R (Abcam ab39398), RANKL (Santa Cruz sc-59982), OPG (Abcam ab65943), BSP (Abcam ab125227), Col-1A (Millipore-Sigma AB765P), DMP1 (Novus Biological NRP1-45525), sclerostin (Abcam ab63097), ORP150 (Santa Cruz sc-398224), FGF23 (R&D Systems MAB2628), IlL-6 (Santa Cruz sc-7920), β-actin (Abcam ab49900) and GAPDH (Cell Signaling Technology 3683). Secondary antibody-HRP conjugates were used an anti-rabbit Alexa488 (Invitrogen A11008). IGF1 ELISA assay. Serum IGF1 was measured using an ELISA kit for murine IGF1 (R&D system MG100) according to the manufacturer's instructions.

Supplemental References:

Guo D, Keightley A, Guthrie J, Veno PA, Harris SE, Bonewald LF. Identification of osteocyte-selective proteins. Proteomics. 2010 Oct;10(20):3688-98. doi: 10.1002/pmic.201000306. PMID: 20845334

Robling AG, Bonewald LF. The Osteocyte: New Insights. Annu Rev Physiol. 2020 Feb 10;82:485-506. doi: 10.1146/annurev-physiol-021119-034332. PMID: 32040934

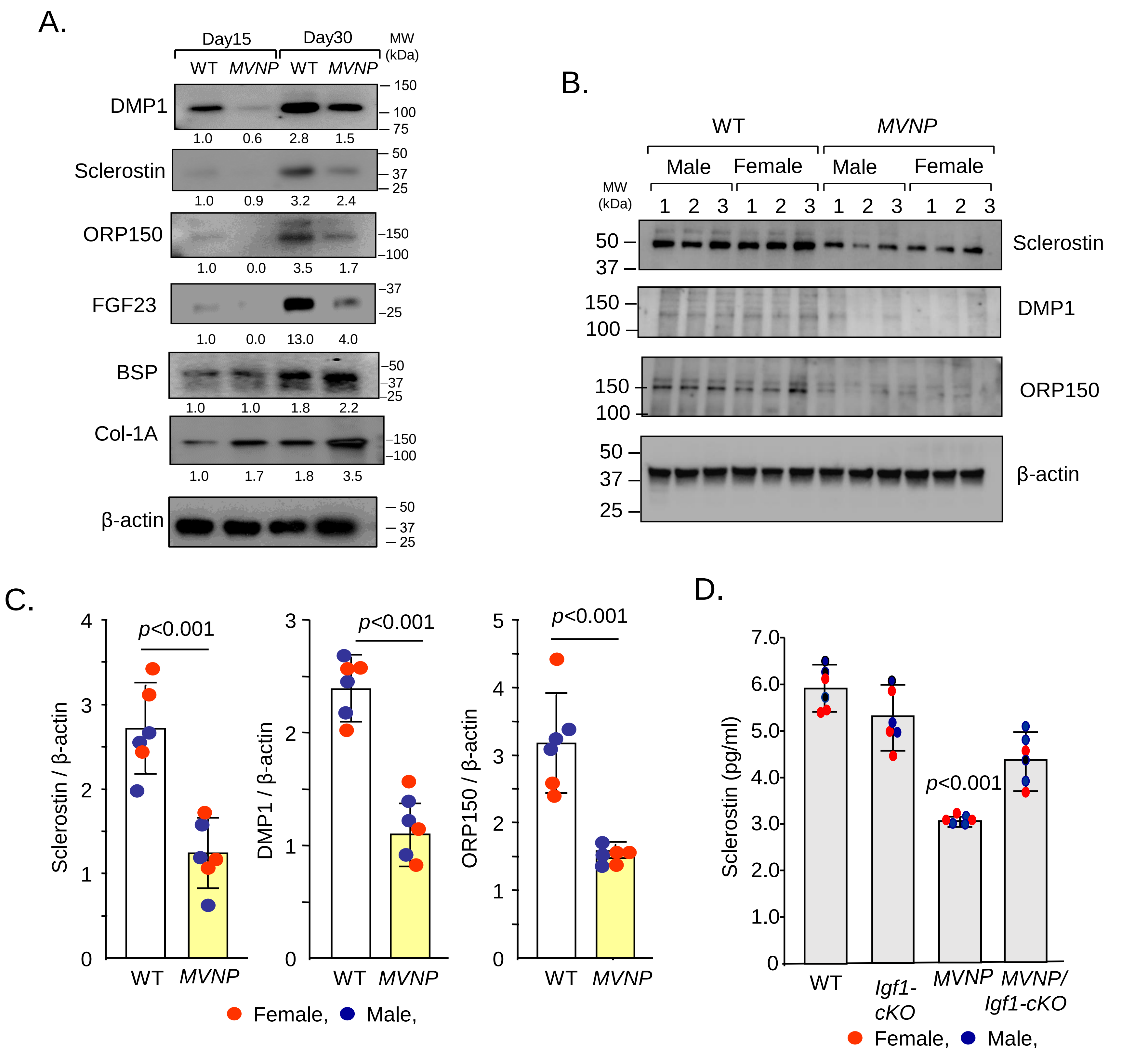

Supplemental Figure 1. The Characteristics of isolated OBs and OCys derived from bone outgrowth cells of 20 months old WT and *MVNP* mice. (**A**) The expression of OB (day 15 culture) and OCy (day 30 culture) markers in outgrowth cells. 1×10^5 cells /well were cultured with 10%FCS in αMEM for 72 hours. Cell lysates were collected with RIPA buffer. Protein expression was visualized by Western blotting using anti-DMP1, anti-sclerostin, anti-ORP150, anti-FGF23, anti-BSP and anti-Col-1A antibodies. β-actin was the loading control. (**B**) OCy markers in 30-day outgrowth cultures of bones from male and female WT and *MVNP* mice grown as in A. (**C**) Expression ratios for each protein/β-actin were quantitated by densitometric scanning the blots shown in B and analyzing with ImageJ software (NIH). This experiment was performed three times with different biological replicates. The results show mean ± SEM; red = female, and blue = male, analyzed by Mann-Whitney U-test. No statistical difference between male and female. (**D**) Sclerostin in conditioned media. OCy-like cells (1×10^5 cells /ml) from 30-day outgrowth cells were cultured for 72 hours and assayed by ELISA. The results suggest that the 15-day outgrowth cells retain osteoblastic characteristics, while the 30-day ones display a more osteocytic phenotype

Supplemental Figure 2

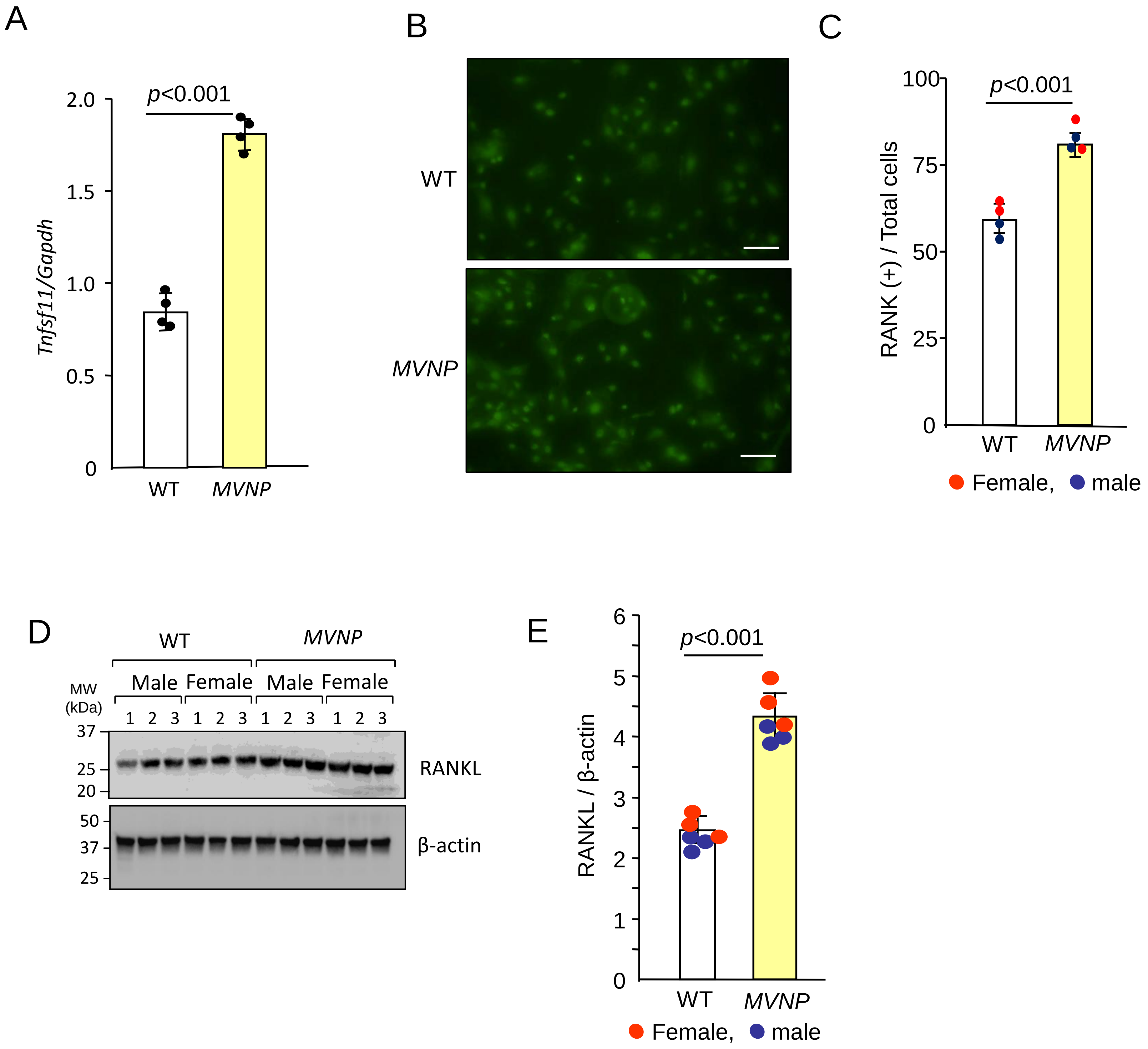

Supplemental Figure 2. RANKL mRNA and protein from primary OCys and OCy-Like cells. (**A**) *RANKL (Tnfsf11)* mRNA in primary OCys isolated by collagenase digestion form long bones of WT and *MVNP* 18-month-old and RNA isolated from 2×10^6 primary OCys. *Tnfsf11* expression was measured by TaqManTM PCR as describe in Methods. Data were shown the mean ± SEM (4 technical replicates from these mice) analyzed by Mann-Whitney U-test. (**B**) RANKL expression in primary OCys. Primary OCys were stained with anti-RANKL antibody and examined by immunofluorescence microscopy. Scale bars;10µm. (**C**) Percent of cells expressing RANKL: RANKL-positive/total OCys in equal squares (700×1050 µm^2) from B. Representative data for male- (blue) and female- (red) derived cells, analyzed as in A. No statistical difference between male and female in each group. (**D**) RANKL protein in OCy-like cells from bone. 1×10^5 cells /ml were cultured with 10%FCS in αMEM for 72 hours and Western blotted using anti-RANKL antibody. β-actin was used as loading control. (**E**) Quantification of D, analysis as In C. There was no statistical difference between male and female groups.

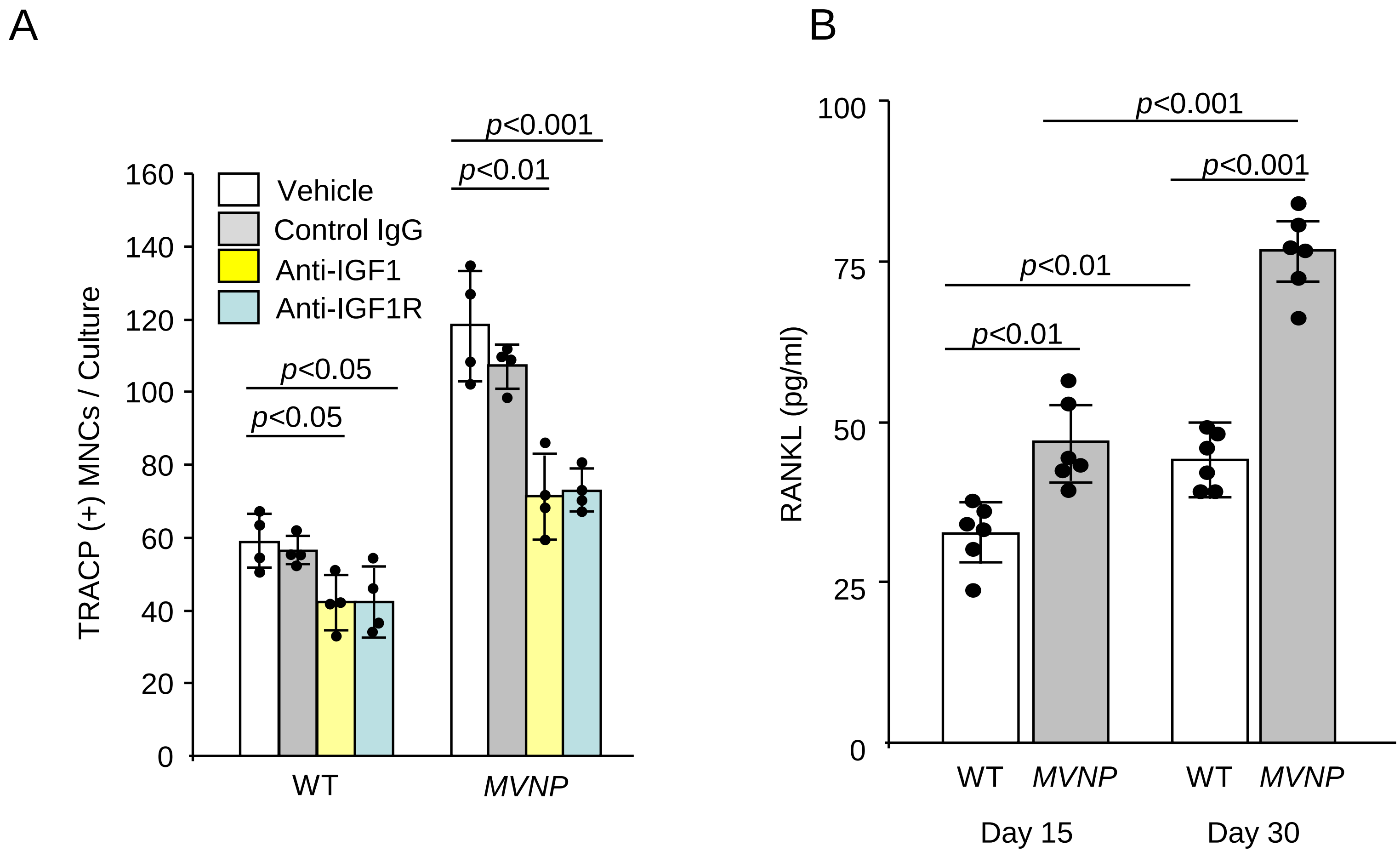

Supplemental Figure 3. (**A**) Autocrine stimulation of OCL formation by IGF1. OCL-precursors from CD11b-positive osteoclast precursors from 22-month-old male WT and *MVNP* mice ($5x10^4$ cells/well; 96 well plate) were treated with M-CSF (10 ng/ml) for 72 hours and cultured with RANKL (50 ng/ml) plus vehicle, rabbit IgG (20 ng/ml), anti-IGF1 (10 µg/ml) or anti-IGF1-receptor (0.5 µg/ml) for 72 hours. The cells were then stained for TRACP. The results were showed the mean ± SEM (n=4). The data were analyzed using a 1-way ANOVA with Tukey test. NS; not significant different. The assay was performed in triplicate and with cells from female mice; results were similar. (**B**) RANKL in conditioned media from OBs and OCy-like cells. Outgrowth cells ($1×10^5$ cells/ml; 12 well plate) were as in Supplemental Figure 1, cultured for 72 hours and conditioned media collected. RANKL ELISA results are shown as mean ± SEM (n=6) analyzed as in (A). The assay was performed on 2 biological replicates with similar results.

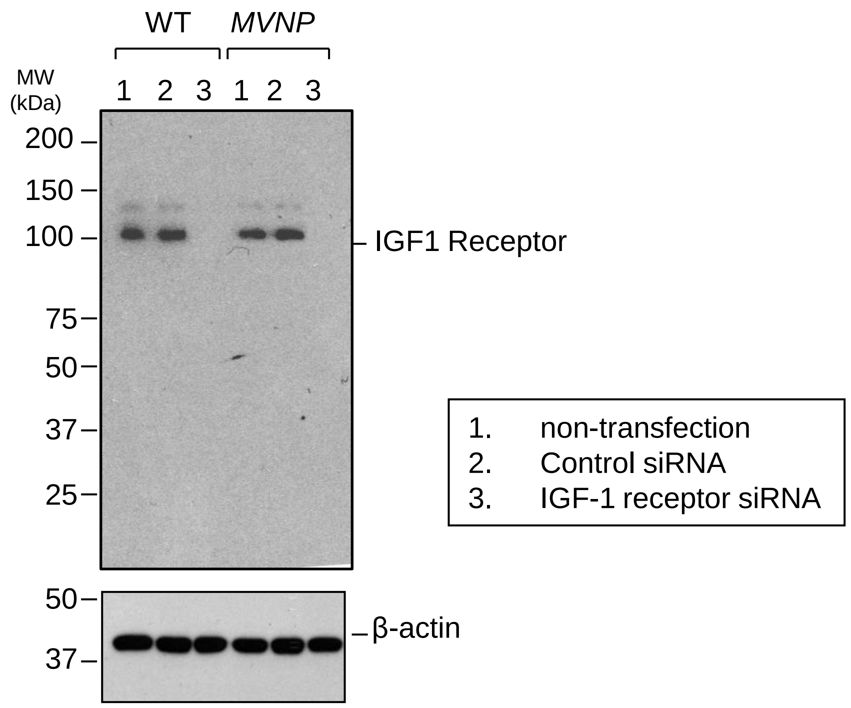

Supplemental Figure 4. Knockdown of IGF1 receptor. OCy-like cells from 18-month-old WT or *MVNP* mice were treated over 48hrs with 100nM control or mouse IGF1 receptor-specific siRNA (Cell Signaling, #6568 or #12482). IGF1R expression was detected by Western blotting using rabbit anti-IGF1R antibody (Cell Signaling, D23H3,) as described in Methods. The experiment was performed three times using different biological replicates with similar results.

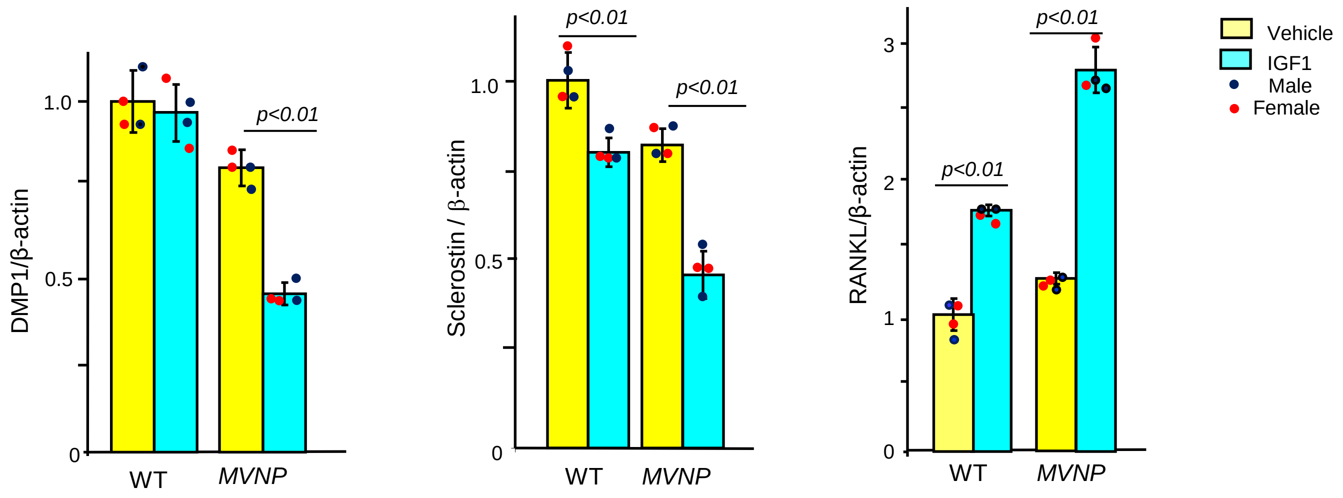

Supplemental Figure 5. IGF1 reduced DMP1 and sclerostin and increased RANKL in osteocyte-like cells. OCy-like cells ($1x10^4$/well) derived from bone in WT and *MVNP* mice at 15-19 months old were cultured with 10% FCS in αMEM for 3 days, then treated ± 10 ng/ml IGF1 in αMEM + 2% FCS for 3 days. Cell lysates were collected with RIPA buffer and Western blotted using anti-DMP1, anti-sclerostin, anti-RANKL, and anti-β-actin as loading control. The blots were analyzed as in Supplemental Figure 1, with n=4 and plotted with normalization to the level in vehicle-treated WT OCy-like cells as 1. The assay was performed three times using different biological replicates, with similar results.

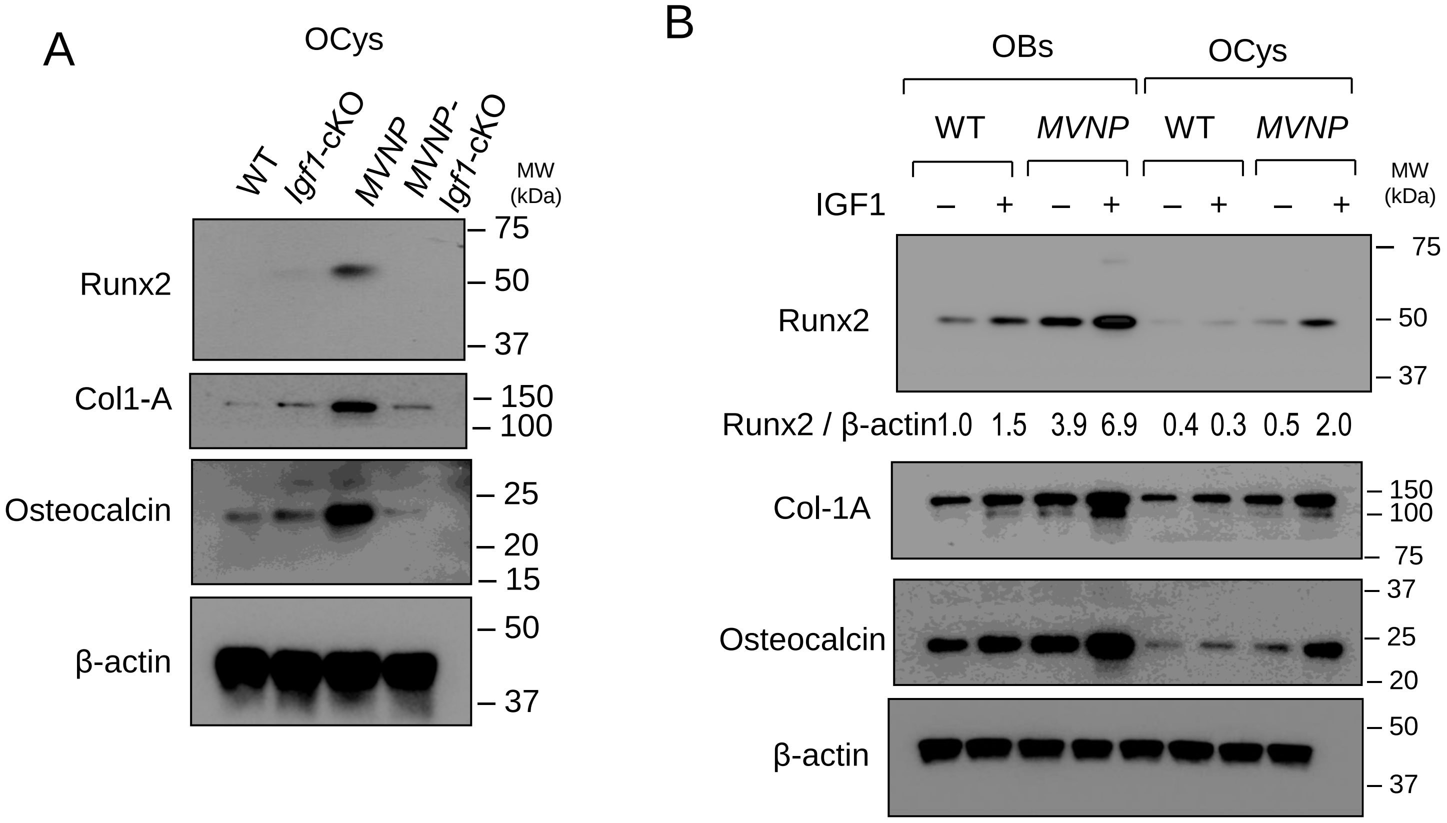

Supplemental Figure 6. Expression of Runx2 by bone cells derived from 20-month-old WT, *Igf1-cKO*, *MVNP* and *MVNP/Igf1-cKO* mice. (**A**) OCy-like cells (1×10^5 cells/well, from day 30 cultures) were grown in αMEM + 10% FCS for 72 hours, then cell lysates were collected with RIPA buffer. The expressions of Runx2, Col-1A and osteocalcin were measured by Western blotting using anti-Runx2, anti-Col-1A and anti-osteocalcin antibodies. β-actin was used as loading control. (B) Runx2 responses to IGF1. Cells from WT or *MVNP* mice as in A from 15-day and 30-day cultures were treated with IGF-1, and Western blots were analyzed for Runx2 as in Supplemental Figure 1.

A.

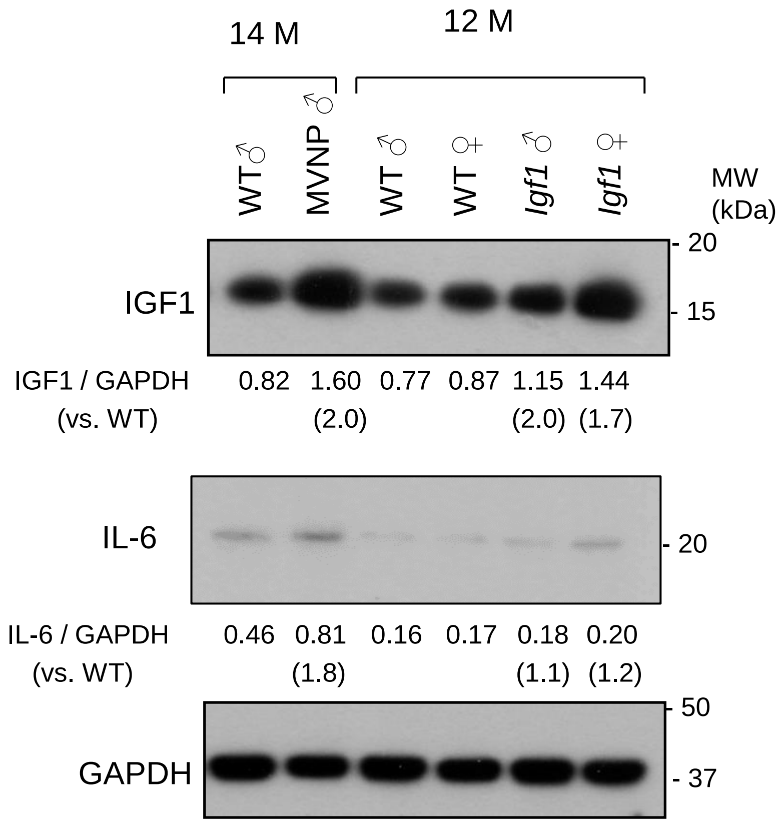

B.

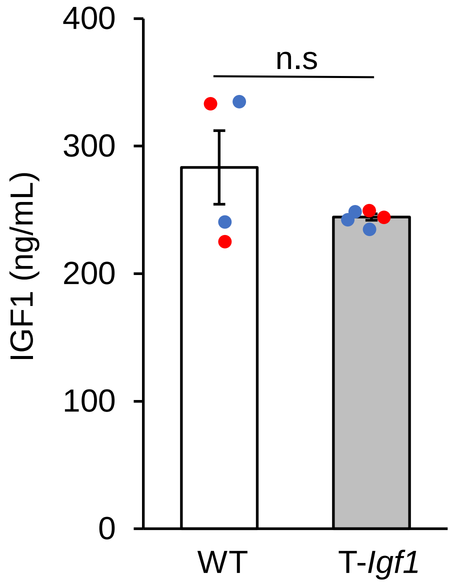

Supplemental Figure 7 (A) IGF1 and IL-6 expression in OCLs formed in mouse BM cultures. IGF1 and IL-6 expression was assayed by Western blotting using anti–IGF1 or -IL-6 antibodies as described in Methods. GAPDH was used as the loading control. The expression levels of IGF1 and IL-6 were quantitated by ImageJ software. The basal ratio for each protein/loading control for OCLs from WT mice cocultured with WT-OCLs was set at 1.0. (B) Serum IGF1 in 16 months of age of T-*Igf1* and WT mice. ELISA assay for IGF1 was described in Methods. Results are expressed as the mean ± SEM for WT (2 male, 2 female, 16 months) and T-Igf1 mice (3 male, 2 female, 16 months). The data were analyzed using a 1-way ANOVA with Tukey test. The blue circles represent results from male mice; red circles represent results from female mice.
